# Discordance between LDL-C and Apolipoprotein B Levels and Its Association with Renal Dysfunction: Insights from a Population-Based Study

**DOI:** 10.3390/jcm11020313

**Published:** 2022-01-09

**Authors:** Mohsen Mazidi, Richard J. Webb, Gregory Y. H. Lip, Andre P. Kengne, Maciej Banach, Ian G. Davies

**Affiliations:** 1 Nuffield Department of Population Health, University of Oxford, Richard Doll Building, Old Road Campus, Oxford OX3 7LF, UK; 2 Department of Twin Research & Genetic Epidemiology, Kings College London, 4th Floor, South Wing St Thomas’, London SE1 7EH, UK; 3 Faculty of Science, Hope Park Campus, School of Health Sciences, Liverpool Hope University, Taggart Avenue, Liverpool L16 9JD, UK; webbr1@hope.ac.uk; 4 Liverpool Centre for Cardiovascular Science, University of Liverpool and Liverpool Heart & Chest Hospital, Liverpool L14 3PE, UK; gregory.lip@liverpool.ac.uk; 5 Aalborg Thrombosis Research Unit, Department of Clinical Medicine, Aalborg University, DK-9100 Aalborg, Denmark; 6 Non-Communicable Diseases Research Unit, South African Medical Research Council, Cape Town 7505, South Africa; Andre.Kengne@mrc.ac.za; 7 Cardiovascular Research Centre, University of Zielona Gora, 65-046 Zielona Gora, Poland; maciej.banach@umed.lodz.pl; 8 Department of Preventive Cardiology and Lipidology, Medical University of Lodz (MUL), 93-338 Lodz, Poland; 9 Research Institute of Sport and Exercise Science, Liverpool John Moores University, Liverpool L3 3AF, UK

**Keywords:** low-density lipoprotein cholesterol, apolipoprotein B, chronic kidney disease, epidemiology, NHANES, discordance

## Abstract

Low-density lipoprotein cholesterol (LDL-C) and apolipoprotein B (ApoB) are established markers of atherosclerotic cardiovascular disease (ASCVD), but when concentrations are discordant ApoB is the superior predictor. Chronic kidney disease (CKD) is associated with ASCVD, yet the independent role of atherogenic lipoproteins is contentious. Four groups were created based upon high and low levels of ApoB and LDL-C. Continuous and categorical variables were compared across groups, as were adjusted markers of CKD. Logistic regression analysis assessed association(s) with CKD based on the groups. Subjects were categorised by LDL-C and ApoB, using cut-off values of >160 mg/dL and >130 mg/dL, respectively. Those with low LDL-C and high ApoB, compared to those with high LDL-C and high ApoB, had significantly higher body mass index (30.7 vs. 30.1 kg/m^2^) and waist circumference (106.1 vs. 102.7 cm) and the highest fasting blood glucose (117.5 vs. 112.7 mg/dL), insulin (16.6 vs. 13.1 μU/mL) and homeostatic model assessment of insulin resistance (5.3 vs. 3.7) profiles (all *p* < 0.001). This group, compared to those with high LDL-C and high ApoB, also had the highest levels of urine albumin (2.3 vs. 2.2 mg/L), log albumin-creatinine ratio (2.2 vs. 2.1 mg/g) and serum uric acid (6.1 vs. 5.6 mg/dL) and the lowest estimated glomerular filtration rate (81.3 vs. 88.4 mL/min/1.73 m^2^) (all *p* < 0.001). In expanded logistic regression models, using the low LDL-C and low ApoB group as a reference, those with low LDL-C and high ApoB had the strongest association with CKD, odds ratio (95% CI) 1.12 (1.08–1.16). Discordantly high levels of ApoB are independently associated with increased likelihood of CKD. ApoB remains associated with metabolic dysfunction, regardless of LDL-C.

## 1. Introduction

Low-density lipoproteins (LDLs) are implicated in the pathogenesis of atherosclerotic cardiovascular disease (ASCVD) [1]. This truism appears to be driven by plasma levels of apolipoprotein B (ApoB), a proteinaceous molecule present on all atherogenic particles, including LDL [2]. ApoB is not only causal of ASCVD but is also a superior risk marker than the cholesterol content of the LDL particles themselves [2,3]. In addition, a discordance between levels of ApoB and LDL-C predisposes individuals to either an increased or decreased risk of ASCVD, compared to those with concordant levels of the two markers [4].

Individuals with chronic kidney disease (CKD) often present with dyslipidaemia, consisting of decreased levels of high-density lipoprotein cholesterol (HDL-C), hypertriglyceridemia, together with raised levels of ApoB and varying levels of LDL-C [5,6]. This abnormal phenotype, which appears to be largely driven by ApoB, is thought to be mediated via the disrupted production and clearance of atherogenic lipoproteins, leading to an increased plasma residence time [7]. This exacerbates their oxidation and glycation, promotes systemic inflammation, and activates deleterious cell signalling pathways, leading to a milieu which ultimately contributes to a decline in kidney function [7,8].

The variability of LDL-C in this pathology may indicate that the level of discordance between ApoB and LDL-C is an important contributor to CKD [6]. However, the independent role of atherogenic lipoproteins in the development of CKD remains contentious, with some studies showing associations, whereas others have not [9,10,11].

In this study, we aimed to investigate, for the first time, the association between ApoB and LDL-C and their discordance with tests of renal function and risk of CKD. We tested the hypothesis that ApoB and LDL-C discordance is associated with CKD. We used data from a nationally representative sample, the National Health and Nutrition Examination Survey (NHANES).

## 2. Design and Methods

### 2.1. Study Population

This cross-sectional study used data derived from the US NHANES. Ethical approval for the underlying protocol was obtained from The National Center for Health Statistics (NCHS) Research Ethics Review Board [12]. Written informed consent was obtained from all participants and the study complied with the 1975 Declaration of Helsinki for medical research involving human subjects.

The current study used data from the two 2-year NHANES survey cycles obtained between 2005 and 2012, restricted to participants aged ≥ 18 years. Details on NHANES laboratory and medical technologists’ procedures and anthropometry procedures are described elsewhere [13]. Briefly, a blood sample was drawn from the participants’ antecubital vein. ApoB was measured using an immunochemical assay and LDL-C was measured enzymatically using a Roche Modular P Chemistry Analyzer [14,15]. Poverty-to-income index was measured using the Department of Health and Human Services’ (HHS) poverty guidelines. Smoking status was self-reported, and participants were classified as current smoker or not. The DxC800 modular chemistry side used the Jaffe rate method (kinetic alkaline picrate) to determine the concentration of creatinine in serum. The creatinine calibration is traceable to an isotope dilution mass spectrometry reference method [16]. Urinary creatinine (assessed by the Jaffe rate reaction) and urinary albumin (assessed by a solid-phase fluorescent immunoassay from a random urine sample) [17] were used to calculate albumin-to-creatine ratio (ACR). The CKD Epidemiology Collaboration (CKD-EPI) equation was used to calculate estimated glomerular filtration rate (eGFR) in ml/min/1.73 m^2^ and eGFR lower than 60 mL/min per 1.73 m^2^ was used to define low eGFR. ACR > 30 mg/g was used to define albuminuria, and the presence of either low eGFR or albuminuria was used to define CKD in line with Kidney Disease: Improving Global Outcomes (KDIGO) 2012 recommendations [18]. Details on recording dietary intake have been previously described [19].

### 2.2. Statistical Analysis

Analyses were conducted according to the guidelines of the Centers for Disease Control and Prevention for analysis of the NHANES dataset, accounting for masked variance and using their suggested weighting methodology [20]. High levels of LDL-C and ApoB were defined by cut-off values of >160 mg/dL and >130 mg/dL, respectively, which are regarded as thresholds for higher risk of ASCVD as determined by expert consensus [21], which resulted in four concordant/discordant groups: group 1 (low LDL-C, low ApoB), group 2 (low LDL-C, high ApoB), group 3 (high LDL-C, low ApoB) and group 4 (high LDL-C, high ApoB). Continuous and categorical demographic variables were compared across the four groups using analysis of variance (ANOVA) and chi-square tests, respectively. Adjusted means of kidney function markers (serum creatinine, ACR, eGFR and serum uric acid) based on LDL-C and ApoB concordant/discordant categories were compared using analysis of covariance (ANCOVA). These models were adjusted for age, sex, ethnicity, poverty-to-income ratio, fasting blood glucose, systolic and diastolic blood pressure, body mass index (BMI, kg/m^2^), diabetes mellitus (DM) (self-reported history of DM or fasting plasma glucose ≥126 mg/dL) and hypertension (HTN, diagnosed in individuals with systolic blood pressure ≥140 mmHg, a diastolic blood pressure ≥90 mmHg or in those on antihypertensive drugs) [22].

Logistic regression models, with 3 different levels of adjustments (model 1: age, sex, ethnicity and poverty-to-income ratio; model 2: as model 1, but additionally adjusted for fasting blood glucose, systolic and diastolic blood pressure and HTN; model 3: as model 2, but additionally adjusted for DM, BMI and CRP), were used to derive the odds ratio (OR) and 95% confidence interval (CI) for the association with prevalent CKD based on LDL-C and ApoB concordant/discordant categories, always using group 1 (low LDL-C, low ApoB) as reference. A *p*-value < 0.05 was used to determine statistical significance.

## 3. Results

A total of 13,767 participants (with mean age 47.3 y; 48.5% being male) were included in the current analysis; 6.7% had prevalent CKD. Table 1 shows the demographic and clinical characteristics of the study population based on LDL-C and ApoB concordant/discordant categories. Overall mean body mass index (BMI) and waist circumference (WC) were 28.8 kg/m^2^ and 98.7 cm, respectively.

Significant differences were apparent for all continuous and categorical demographic variables across the four groups (all *p* < 0.001). The subjects with low LDL-C and high ApoB (group 2), when compared to those with high LDL-C and high ApoB (group 4), had significantly higher BMI (30.7 ± 0.6 and 30.1 ± 0.4 kg/m^2^, respectively) and WC (106.1 ± 1.7 and 102.7 ± 1.3 cm, respectively) (both *p* < 0.001). Subjects with low LDL-C and high ApoB (group 2) also had the highest insulin and glucose levels compared to those with high LDL-C and high ApoB, including fasting blood glucose (FBG) (117.5 ± 6.0 and 112.7 ± 3.2 mg/dL, respectively), insulin (16.6 ± 1.4 and 13.1 ± 1.0 μU/mL, respectively) and homeostatic model assessment of insulin resistance (HOMA-IR) (5.3 ± 0.9 and 3.7 ± 0.3, respectively) (all *p* < 0.001, Table 1).

Adjusted (age, sex, ethnicity, fasting blood glucose, systolic and diastolic blood pressure, BMI, DM and HTN) mean levels of kidney function markers by LDL-C and ApoB concordant/discordant categories are shown in Table 2. Subjects within group 2 (Low LDL-C, High ApoB) had higher levels of urine albumin compared to group 4 (High LDL-C, High ApoB) (2.3 ± 0.0 and 2.2 ± 0.0 mg/L, respectively), as well as higher levels of ACR (2.2 ± 0.0 and 2.1 ± 0.0 mg/g, respectively) and serum uric acid (6.1 ± 0.3 and 5.6 ± 0.2 mg/dL, respectively) (all *p* < 0.001). The Low LDL-C, High ApoB group also had significantly lower levels of eGFR (81.3 ± 0.5 mL/min/1.73 m^2^) compared with other groups, even group 4 (High LDL-C, High ApoB) (88.4 ± 0.4 mL/min/1.73 m^2^, *p* < 0.001, Table 2)

As shown in Table 3, three different models were used with a wide range of potential confounders to evaluate the odds of CKD based on the LDL-C and ApoB concordant/discordant categories. In the model adjusted for age, sex, ethnicity and poverty-to-income ratio compared with group 1 (Low LDL-C, Low ApoB), the OR (95% CI) for CKD was 1.09 (1.06–1.11 for group 3 (High LDL-C, Low ApoB); 1.18 (1.10–1.34) for group 4 (High LDL-C, High ApoB) and 1.80 (1.25–1.90) for group 2 (Low LDL-C, High ApoB). In the expanded models with further adjustment for age, sex, ethnicity, poverty-to-income ratio, fasting blood glucose, systolic and diastolic blood pressure, HTN, DM and CRP, similar associations were observed, i.e., group 2 (Low LDL-C, High ApoB) had the highest (1.12 (1.08–1.16), Table 3) likelihood of CKD compared to group 1. Moreover, group 2 (Low LDL-C, High ApoB) also had the highest likelihood of albuminuria (1.08 (1.03, 1.13)) compared with other groups.

## 4. Discussion

The current study aimed to determine the association of discordant ApoB and LDL-C with CKD. Our principal novel finding is that those individuals in group 2 (Low LDL-C, High ApoB) were at an increased likelihood of prevalent CKD, even when accounting for a range of potentially confounding variables. Second, this group also presented the lowest glomerular filtration rate as well as the highest log albumin-creatinine ratio and serum uric acid. Third, these subjects had the greatest metabolic disruption, including the highest levels of plasma triglycerides, glucose and insulin, as well as raised mean blood pressure, waist circumference, BMI and CRP, together with the lowest levels of HDL-C.

The findings from groups 2 and 4 reflect the so-called ‘atherogenic dyslipidaemia’, a trait which is predominantly characterised by decreased levels of HDL-C, moderately raised levels of triglycerides and a shift towards smaller LDL particles, an attribute likely demonstrated in group 2, whereby the LDL particles may be depleted of cholesterol [23]. Although this profile was most pronounced in group 2, it is noteworthy that group 4 (High LDL-C, High ApoB) also presented a poor metabolic profile. Despite group 2 presenting with the most discordance between ApoB and LDL-C, which is indicative of the greatest risk of ASCVD [4], the total overall ApoB level was lower than that found in group 4 (High ApoB, High LDL-C). The presence of this atherogenic phenotype, together with an excess of ectopic fat, has been associated with increased systemic oxidation, chronic low-grade inflammation and hyperinsulinemia, which concomitantly creates an environment conducive to a markedly increased risk of ASCVD [24].

The evidence suggests that several attributes implicated in this process may ultimately lead to nephron damage. For example, hyperlipidaemia has been associated with an increased production of reactive oxygen species, which can lead to their perpetual regeneration and deleterious modifications to ApoB-containing lipoproteins, resulting in increased oxidative stress and subsequent glomerulosclerosis and tubulointerstitial injury [25,26]. Moreover, a decrease in the size of HDL particles results in an impairment of their normal antioxidant, anti-inflammatory and reverse cholesterol transport activities, serving to further exacerbate the situation [27]. The culmination of this has been highlighted in several studies demonstrating that patients with lipid abnormalities are at an increased risk of end-stage renal failure and are ultimately more likely to require renal replacement therapy [10,28].

Likewise, CRP, although not causally related to ASCVD, is generally regarded as a useful adjunctive biomarker and has been implicated in renal dysfunction via multiple mechanisms, including via the promotion of local inflammation and fibrosis of injured renal tissue [29]. Similarly, glucose homeostasis is another key component implicit in the pathogenesis of ASCVD, yet also implicated in the development of CKD, with hyperinsulinemia being shown to promote the uptake of serum uric acid by the kidneys and coexisting with hypertension to further accelerate renal damage [30,31,32].

In the present study, groups 2 and 4 reveal a predominance of dyslipidaemia, poor glycaemic control, and adiposity. It is therefore reasonable to suggest that these aspects may predispose individuals in these groups to have decreased kidney function. Indeed, group 2 displayed particularly compromised renal function, although this did not track with ApoB, which was lower in group 2 than in group 4. Despite this, the levels of discordance between ApoB and LDL-C is a driving force for ASCVD risk, and this is applicable to kidney dysfunction [4]. Indeed, our findings show that when considering CKD, group 2 appears to have the highest odds of developing the disease. Furthermore, this remains the case even when inflammation (CRP) has been adjusted for. This suggests that the impact of ApoB upon CKD may even exceed that of chronic inflammation, a known factor which frequently accompanies the disease and a finding also shown by others [11,33].

### Limitations and Strengths

Despite our study using data from the NHANES survey, which is nationally representative of the American population, there are several limitations which should be recognised. For example, the cut-off values we chose to use for LDL-C and ApoB are based upon expert consensus regarding ASCVD in the general population, as opposed to in patients with CKD where consensus regarding ApoB levels has not yet been reached [21]. Furthermore, whereas other studies investigating the impact of discordance between ApoB, and LDL-C have opted to use median values, which may reveal further differences between the groups [21]. It is also crucial to recognise that the NHANES study is cross-sectional and therefore does not give any insights into the temporal relationship between exposures and outcomes, therefore warranting further prospective cohort studies. Finally, the use of logistic regression, although offering insights into the relationship between ApoB and LDL-C and CKD, is not immune to residual confounding, producing spurious correlations and suffering from multicollinearity, as well as being unable to distinguish between reverse causality.

## 5. Conclusions

In summary, the novel findings from our study are that discordantly high levels of ApoB in relation to LDL-C are related to an increased likelihood of prevalent CKD, even in the presence of inflammation, diabetes, hypertension, and a range of other predictors. Additionally, regardless of LDL-C levels, ApoB remains associated with metabolic dysfunction, with individuals presenting with discordantly high levels of ApoB having the highest likelihood of CKD. Our findings therefore suggest that the measurement of ApoB should potentially be considered, especially for patients with atherogenic dyslipidaemia, since ApoB is more likely to be discordantly high in relation to LDL-C.

## Figures and Tables

**Table 1:** Demographic and clinical characteristics of the total population based on LDL-C and ApoB categories.

Characteristics		Group 1 (Low LDL-C, Low ApoB) (*n* = 11,956) (Reference Group)	Group 2 (Low LDL-C, High ApoB) (*n* = 274)	Group 3 (High LDL-C, Low ApoB) (*n* = 698)	Group 4 (High LDL-C, High ApoB) (*n* = 839)	*p*-Value
Age (Years)		48.7 ± 1.5	55.5 ± 1.3	51.0 ± 1.8	56.0 ± 1.6	<0.0001
Sex (%)	Male	49.3	51.5	52.2	45.0	<0.0001
	Female	50.7	49.5	47.8	55.0	
Anthropometric Parameters	BMI (kg/m^2^)	28.2 ± 0.2	30.7 ± 0.6	28.2 ± 0.5	30.1 ± 0.4	<0.0001
	WC (cm)	97.2 ± 1.0	106.1 ± 1.7	96.7 ± 1.4	102.7 ± 1.3	<0.0001
Insulin and Glucose Parameters	Fasting blood glucose (mg/dL)	101.2 ± 1.1	117.5 ± 6.0	99.4 ± 2.6	112.7 ± 3.2	<0.0001
	Plasma insulin (μU/mL)	13.2 ± 0.2	16.6 ± 1.4	11.4 ± 0.8	13.1 ± 1.0	<0.0001
	HOMA-IR	3.5 ± 0.1	5.3 ± 0.9	2.8 ± 0.1	3.7 ± 0.3	<0.0001
Serum CRP (mg/dL)		0.4 ± 0.01	0.5 ± 0.1	0.3 ± 0.0	0.5 ± 0.1	<0.0001
SBP (mmHg)		121.5 ± 0.4	131.1 ± 3.0	126.5 ± 2.2	129.4 ± 1.6	<0.0001
DBP (mmHg)		67.9 ± 0.3	71.5 ± 2.3	70.2 ± 1.5	72.9 ± 1.0	<0.0001
Total cholesterol (mg/dL)		184.6 ± 0.7	240.1 ± 3.0	251.1 ± 1.9	282.2 ± 2.4	<0.0001
HDL-C (mg/DL)		54.2 ± 0.4	43.0 ± 2.0	56.6 ± 1.4	51.5 ± 1.2	<0.0001
LDL-C (mg/dL)		105.3 ± 0.6	144.3 ± 3.0	170.5 ± 1.0	192.1 ± 2.4	<0.0001
ApoB (mg/dL)		87.0 ± 1.0	138.0 ± 1.0	118.6 ± 1.0	145.4 ± 0.9	<0.0001
Triglycerides (mg/dL)		116.1 ± 1.9	233.0 ± 120.1	117.1 ± 5.4	176.7 ± 6.2	<0.0001
TG/HDL ratio		2.4 ± 0.0	5.9 ± 0.4	2.3 ± 0.2	3.7 ± 0.2	<0.0001
LDL-C/ApoB ratio		1.3 ± 0.0	1.0 ± 0.0	1.6 ± 0.0	1.3 ± 0.0	<0.0001
Non-HDL-C		131.0 ± 0.9	196.6 ± 3.0	194.6 ± 1.3	229.9 ± 2.8	<0.0001

Values expressed as a mean and SEM or percent. Abbreviations: ApoB, apolipoprotein B; BMI, body mass index; CRP, C-reactive protein; DBP, diastolic blood pressure; HDL-C, high-density lipoprotein cholesterol; HOMA-IR, Homeostatic Model Assessment of Insulin Resistance; LDL-C, low-density lipoprotein cholesterol; SBP, systolic blood pressure; TG, triglycerides and WC, waist circumference.

**Table 2:** Age, sex, race, fasting blood glucose, systolic and diastolic blood pressure, body mass index, diabetes and hypertension-adjusted mean of markers of kidney function based on LDL-C and ApoB concordant/discordant categories.

Variables	ApoB and LDL-C Categories				
	Group 1 (Low LDL-C, Low ApoB) (*n* = 11,956)	Group 2 (Low LDL-C, High ApoB) (*n* = 274)	Group 3 (High LDL-C, Low ApoB) (*n* = 698)	Group 4 (High LDL-C, High ApoB) (*n* = 839)	*p*-Value
Serum Creatinine (mg/dL)	0.8 ± 0.0	0.8 ± 0.0	0.8 ± 0.0	0.8 ± 0.0	0.312
Log Urinary Albumin (mg/L)	1.9 ± 0.0	2.3 ± 0.0	2.1 ± 0.0	2.2 ± 0.0	0.009
Glomerular filtration rate (mL/min/1.73 m²)	97.2 ± 0.4	81.3 ± 0.5	90.4 ± 0.5	88.4 ± 0.4	<0.001
Log Albumin-Creatinine Ratio (mg/g)	2.0 ± 0.0	2.2 ± 0.0	2.1 ± 0.0	2.1 ± 0.0	<0.001
Serum Uric acid (mg/dL)	5.0 ± 0.2	6.1 ± 0.3	5.1 ± 0.1	5.6 ± 0.2	<0.001

Values expressed as estimated mean and standard error. Variables were compared based on LDL-C and ApoB concordant/discordant categories using analysis of co-variance (ANCOVA) test.

**Table 3:** Adjusted logistic regression to examine the association between LDL-C and ApoB concordant/discordant categories and chronic kidney disease (CKD).

	**Likelihood of CKD with Different Models**					
**Variables**	Age, Sex, Race and Poverty-to-Income Ratio		Age, Sex, Race, Poverty-to-Income Ratio, Alcohol Intake, Energy Intake, Smoking, Physical Activity, Fasting Blood Glucose, Systolic and Diastolic Blood Pressure, HTN and DM		Age, Sex, Race, Poverty-to-Income Ratio, Alcohol Intake, Energy Intake, Smoking, Physical Activity, Fasting Blood Glucose, Systolic and Diastolic Blood Pressure, HTN, DM and CRP	
	Odds Ratio	Lower Bound- Upper Bound	Odds Ratio	Lower Bound- Upper Bound	Odds Ratio	Lower Bound- Upper Bound
Group 2 (Low LDL-C, High ApoB) (*n* = 274)	1.80	1.25–1.90	1.20	1.10–1.29	1.12	1.08–1.16
Group 3 (High LDL-C, Low ApoB) (*n* = 698)	1.09	1.06–1.11	1.16	0.96–1.40	1.07	0.96–1.19
Group 4 (High LDL-C, High ApoB) (*n* = 839)	1.18	1.10–1.34	1.14	1.08–1.21	0.96	0.91–1.02

Group 1 (Low LDL-C, Low ApoB) was always used as reference. CKD: chronic kidney disease; HTN: Hypertension; DM: Diabetes mellitus; CRP: C-reactive protein. Shaded boxes indicate that *p* < 0.05.

## Data Availability

All data used in the study are publicly available at: https://www.cdc.gov/nchs/nhanes/index.htm (United States, accessed on 6 November 2021).
